# The effects of detraining and retraining periods on fat-mass and fat-free mass in elite male soccer players

**DOI:** 10.7717/peerj.7466

**Published:** 2019-08-13

**Authors:** Luis Suarez-Arrones, Pilar Lara-Lopez, Rafael Maldonado, Nacho Torreno, Moises De Hoyo, Fabio Yuzo Nakamura, Valter Di Salvo, Alberto Mendez-Villanueva

**Affiliations:** 1 Department of Sport and Informatics, Section of Physical Education and Sport, Pablo de Olavide University, Sevilla, Spain; 2 Performance Department, FC Basel 1893, Basel, Switzerland; 3 Football Science Department, Pablo de Olavide University, Sevilla, Spain; 4 Sports Science Department, FC Girondins de Bordeaux, Bordeaux, France; 5 Universidad de Sevilla, Sevilla, Spain; 6 The College of Healthcare Sciences, James Cook University, Townsville, Australia; 7 Associate Graduate Program in Physical Education UPE/UFPB, João Pessoa, PB, Brazil; 8 Football Performance & Science Department, Aspire Academy, Doha, Qatar; 9 Department of Movement, Human and Health Sciences, University of Rome “Foro Italico”, Rome, Italy

**Keywords:** Football, Power, Inertial devices, Neuromuscular training

## Abstract

The aim of the study was to examine the effects of a detraining period (DTP) (i.e., off-season) with an individually prescribed training program, and a retraining period (RTP) (i.e., pre-season) combining soccer and flywheel-based strength training on fat-free mass (FFM) and fat-mass (FM) in 10 elite professional male soccer players. The present study used a controlled repeated-measures research design to investigate the changes in FFM and FM using dual-energy X-ray absorptiometry. Whole body %FM increased (effect size (ES) = 0.87 ± 0.46) and FFM reduced after DTP (ES = −0.30 ± 0.19), returning to values comparable to the end of the previous season after RTP. At regional levels, arms, legs, and trunk %FM increased (ES = from 0.42 to 1.29) while trunk-FFM was reduced (ES = −0.40 ± 0.26) after DTP, returning to the values observed at the end of the previous season after RTP. Legs-FFM did not change after DTP, with a substantial increase after RTP in comparison with pre-season values (ES = 0.34 ± 0.29 and 0.53 ± 0.36 for the right and left leg, respectively). Despite the small sample size of the present study, the findings indicate that elite soccer players can be allowed 2 weeks of rest during a five-week DTP, since the changes in %FM and FFM were relatively small, and FM and FFM returned to the optimal initial values for competition after the proposed RTP during the pre-season.

## Introduction

Soccer is considered a high-intensity intermittent sport, requiring high levels of physical fitness related to the ability to perform powerful actions such as sprinting, jumping and change of direction, all regarded as important for player competitive success (Reilly, Bangsbo & Franks, 2000). In Serie-A league (i.e., first tier domestic competition in Italy), the whole-season is planned in three different periods: pre-competition (pre-season), competition, and transition (off-season). The transition period in elite professional soccer is a window of opportunity for physical and mental recovery, and to “rebuild” players for the upcoming season (Silva et al., 2016). Generally, this period is characterized by a substantial reduction in training, even including complete training cessation for a few days. During off-season, players also participate in other sport activities to retain fitness and/or follow individualized training programs offered by their clubs to facilitate faster adaptation during the subsequent pre-season phase (Silva et al., 2016). The pre-season is commonly characterized by a high frequency of training sessions and friendly games shortly after returning to training, with rapid increases in training load within a few days. In addition, the commercial obligations of clubs mean that players frequently travel and play under high psychological and physiological stress (Nedelec et al., 2015) combined with accumulation of high training loads and subsequent fatigue (Silva et al., 2016). For this reason, players should start pre-season with optimal fitness levels in order to better tolerate the rapid increases in training load, trying to minimize the risk of injury (Gabbett & Domrow, 2007). Previous studies have shown that reduced lean-mass and muscle strength deficiency constitute the main risk factors for muscle strain injuries in soccer (Croisier et al., 2008; De Hoyo et al., 2014; Mendez-Villanueva et al., 2016; Timmins et al., 2016). Previous studies have shown that a neural factor accounts for the early strength gains occurring during the first weeks of training, while a hypertrophic factor has been claimed to have a later onset (Sale, 1988; Seynnes, Boer & Narici, 2007). The relative increase in muscle strength is greater than what could be accounted for by the increase in muscle volume (Tesch et al., 2004); therefore, optimal lower-limb lean-mass and consequently muscular strength at the beginning of the pre-season might help soccer players to mitigate muscular damage during the rapid increases in training load during pre-season.

Normally, the off-season break negatively influences the body composition of players (Silva et al., 2016). Previous studies with professional soccer players showed increases in the percentage of fat-mass (%FM) (Koundourakis et al., 2014; Reinke et al., 2009; Sotiropoulos et al., 2009) and decreases in lean-mass or fat-free mass (FFM) (Reinke et al., 2009) after the detraining period (DTP), both of which may negatively affect tolerance to high training volumes and intensities during the first weeks of the pre-season, especially when the changes in %FM and FFM are substantial (Silva et al., 2016). During the pre-season, the aim of training should be to improve physical fitness, develop the game model, tactics, and playing strategies to enhance soccer performance (Joo, 2018), and start the in-season period in the best possible condition.

The main goal of strength-training (ST) in soccer is to improve the players’ ability to optimally perform specific and relevant soccer activities inherent to the competitive match (Silva, Nassis & Rebelo, 2015), as well as reduce post-training and post-match markers of muscle damage (Owen et al., 2015) and the risk of injury (Timmins et al., 2016). A recent meta-analysis provided evidence supporting the benefits of flywheel ST to promote skeletal muscle adaptations expressed as strength, power, and muscle size (Maroto-Izquierdo et al., 2017). In this regard, it would make sense to incorporate this ST methodology consistently throughout the soccer season, to enhance muscle adaptations and physical fitness, and reduce the risk of injury in professional players submitted to congested calendars. Based on this, during the detraining period the soccer players should be involved in a strength-training program in order to minimize the lean mass lost, and during all the pre-season in order to return to the previous FFM values (Munguia-Izquierdo et al., 2019; Requena et al., 2017; Suarez-Arrones et al., 2019; Suarez-Arrones et al., 2018b).

To our knowledge, no previous studies have evaluated the effects of a DTP on body composition (BC) data for the whole body as well as regional levels using dual-energy X-ray absorptiometry (DXA) in professional soccer players with extensive experience in eccentric-overload training. In addition, no previous studies have assessed the effects of a retraining period (RTP) of soccer, supplemented with an intensified ST program including flywheel devices, on BC data using DXA. Therefore, the aim of the present study was to examine the effects of five weeks of DTP with an individually prescribed training program, and six and a half weeks RTP (i.e., pre-season) combining soccer and flywheel-based ST on FFM and FM in elite professional male soccer players

## Methods

### Participants

This study involved a group of 10 elite male soccer players belonging to the squad of a Serie A club in Italy qualified to compete in the UEFA Europa League. The mean ± SD age, body mass, and height were 27.3 ± 2.8 years, 78.1 ± 4.6 kg, and 1.83 ± 0.08 m, respectively. Data were collected at the end of the domestic competition, at the beginning of the pre-season, and 6 weeks and a half later during the competition period. To be eligible for the study, players were required to meet the following criteria: (i) have a current professional contract with the first team, (ii) be injury free at the moment of the initial assessment, (iii) have completed >90% of the pre-season training sessions. Players had extensive previous experience in eccentric-overload training using flywheel devices throughout the entire previous season (one or twice a week). Data comes from the periodic monitoring in which players are evaluated over the season, therefore, ethics committee clearance was not required (Winter & Maughan, 2009). Neverless, the study conformed to the recommendations of the Declaration of Helsinki, and the local Institutional Research Ethics Committee (i.e., Qatar Antidoping Lab) approved the investigation and a written informed consent was obtained from the players.

### Experimental design

The present study used a controlled repeated-measures research design to investigate the changes in BC in response to a DTP (with an individually prescribed training program), and after a soccer RTP (pre-season) supplemented with an intensified ST program, in elite male professional players. All players were tested in three different periods; the first assessment took place at the end of the competition period in May (during the final week of the season), the second was performed at the beginning of the pre-season in July to evaluate the DTP (July 6th), and the third was performed during the second week of the competition period to evaluate the RTP (August 23rd). Players were asked to abstain from any physical activity for at least 48 h prior to the experimental testing.

### Anthropometric and body composition analysis

Body mass was measured with an electronic scale (OHAUS Corp., Florham Park, NJ) and stature with a stadiometer (Seca 213, Hamburg, Germany). Body composition (FM and FFM) was assessed by DXA (Hologic QDR Series, Delphi A model, Bedford, MA, USA) using Hologic APEX software, version 13.3:3, and according to the manufacturer’s recommended procedures. Before any measurements, the DXA was calibrated each day with phantoms, as per the manufacturer’s guidelines. The participants assumed a stationary, supine position on the scanning table, with hands level with the hips and feet slightly apart, as in a recent study (Suarez-Arrones et al., 2018b). The athletes removed metal objects or jewellery from their body and wore the same minimal clothing (underwear) for each scan. The athletes were also instructed to follow a standard protocols of food and fluid to ensure hydration was optimal before each scans (Bilsborough et al., 2014). All scanning and analyses were performed by the same operator to ensure consistency and in accordance with standardised testing protocols recognised as best practise (Milsom et al., 2015; Nana et al., 2016; Rodriguez-Sanchez & Galloway, 2015). Whole-body data are reported as total body, excluding the head (Milanese et al., 2015). Scans were performed in the morning before training and fat-free mass and fat-mass along with other parameters were calculated.

### Detraining period (off-season)

The detraining period in the present study consisted of five weeks. During the first two weeks, players were asked to completely rest and avoid any kind of physical activity. Thereafter, for the remaining three weeks, players were instructed to perform an individualized training program that included high-intensity running interval training (HIT) and ST, training 4 days per week. Each training session normally consisted of a warm-up, ST in the gymnasium (gym), and HIT at the end of the session or at a different time of day. The warm-up contained articular mobility and active stretching exercises. The ST was structured into three different session types: core and sensorimotor exercises (12 sessions), functional (exercises involving different joints and planes during specific movements) (three sessions), and structural strength exercises more isolated (three sessions) for upper and lower body using different equipment (free-weight, instability, and suspension training (Kine Dynamic^®^)). The HIT was also organized into three different session types: two sessions of long-interval HIT runs [i.e., 5 × 4 min (∼90% maximal aerobic speed)/3 min rest between repetitions]; 4 sessions of short-interval HIT runs (i.e., 3 × 6 min: 10 s (95% at peak speed reached in the 30–15 Intermittent Fitness Test)/10 s (passive rest)). Furthermore, players performed 2 sessions of repeated-sprint training (i.e., 4 sets of 6 × 40 m shuttle sprints (20 + 20 m)). An example of a one-week individual training programme is shown in Table 1.

**Table 1:** An example of typical training schedule during off-season (detraining period).

**Monday**	**Tuesday**	**Wednesday**	**Thursday**	**Friday**	**Saturday**	**Sunday**
Warm-up (5 min) Articular mobility and active stretching	Warm-up (5 min) Articular mobility and active stretching	Other Sport	Warm-up (5 min) Articular mobility and active stretching	Warm-up (5 min) Articular mobility and active stretching	Off	Off
**Complementary training**	**Complementary training**		**Complementary training**	**Complementary training**		
*Balance + Core + Functional training (Hip focus, 12 exercises (∼20 min):* Frontal Bridge TRX 2 × 6 × 6”; side bridge TRX 2 × 6 × 6” (each side); Bridge 1 leg Fitball 2 × 6 × 6” (each leg); Russian twists on fitball 2 × 30”; single leg balance (closed eyes) on bosu 2 × 30”; single leg jump on bosu 2 × 30”; band adductor 2 × 10 (each leg); barbell glute bridge 2 × 10; standing adductor on fitball 2 × 10 (each side); push up+ side plank 1 leg 2 × 10 (each side); static wall squat 4 × 15”; “the glider” 2 × 10 (each side).	*Balance + Core, 6 exercises (∼10 min):* Frontal Bridge TRX 2 × 6 × 6”; side bridge TRX 2 × 6 × 6” (each side); Bridge 1 leg Fitball 2 × 6 × 6” (each leg); Russian twists on fitball 2 × 30”; single leg balance (closed eyes) on bosu 2 × 30”; single leg jump on bosu 2 × 30”.		*Balance + Core + Hip Muscle, 12 exercises (∼20 min):* Frontal Bridge TRX 2 × 6 × 6”; side bridge TRX 2 × 6 × 6” (each side); Bridge 1 leg Fitball 2 × 6 × 6” (each leg); Russian twists on fitball 2 × 30”; single leg balance (closed eyes) on bosu 2 × 30”; single leg jump on bosu 2 × 30”; band adductor 2 × 10 (each leg); barbell glute bridge 2 × 10; standing adductor on fitball 2 × 10 (each side); push up+ side plank 1 leg 2 × 10 (each side); static wall squat 4 × 15”; “the glider” 2 × 10 (each side).	*Balance + Core, 6 exercises (∼10 min):* Frontal Bridge TRX 2 × 6 × 6”; side bridge TRX 2 × 6 × 6” (each side); Bridge 1 leg Fitball 2 × 6 × 6” (each leg); Russian twists on fitball 2 × 30”; single leg balance (closed eyes) on bosu 2 × 30”; single leg landings on bosu 2 × 30”.		
	**Strength training**			**Strength training**	
	*Upper & Lower body, Circuit training.*3 × 8* exercises (30 min):* leg press 8 rep; push up TRX 8 rep; pull TRX 8 rep; single deadlift with dumbbell 6 rep (each leg); standing dumbbell arm swing 30”; high box jump 6 rep; standing dumbbell fly on fitball 8 rep; barbell lunge split jump 6 rep (each leg).			*Upper & Lower body, Circuit training.*3 × 8* exercises (30 min):* half squat 8 rep, bench press 8 rep, seated cable row 8 rep, barbell curl 8 rep, barbell lunge 8 rep (each side), lateral elevation shoulder 8 rep (each side), pulley triceps extension 8 rep, high box jump 6 rep	
**Endurance training** HIT (long intervals). 2 × [ 3 × 4’ running (800m)] rest/rep: 2’, rest/set: 4’ ****	**Endurance training** HIT (short intervals). 3 × 6’ [20” (98 m): 20” (passive rest)], rest: 3’.		**Endurance training** HIT (short intervals). 3 × 6’ [10” (50 m): 10” (passive rest)], rest/set 4’.	**Endurance training** HIT (short intervals). 8 × 30” running all out straight line, rest: 3’.		


### Retraining period (pre-season)

The pre-season retraining period in the present study lasted six and a half weeks. Players supplemented the soccer training with an ST program structured into four different session types: (i) ST in the gym; (ii) specific ST on the field; (iii) activation training; and (iv) individual training.

ST in the gym was usually organized as circuit training before the soccer drills on the field. Players performed one or two laps of a circuit consisting of 10–12 exercises mainly focusing on the lower-limbs, combining free-weights with non-gravity dependent flywheel inertial devices (Kbox^®^, Yo-Yo technology^®^ and Versa-Pulley^®^) and motorized devices (Exentrix^®^), and including some functional exercises for upper-body and core muscles. In addition, complementary ST sessions were prescribed with exercises for upper-body, core, and lumbo-pelvic stability. ST sessions in the gym lasted 30-40 min each, while complementary sessions lasted 20 min. The main exercises employed in the gym sessions for lower-limbs were as follows: specific soccer movements (side step, cutting, lunge) focusing more on the horizontal force (anterior-posterior/posterior-anterior / lateral and rotational) using Versa Pulley^®^ (VP) (0.19 kg/m^2^ and 0.26 kg/m^2^ inertias), several bilateral and unilateral half-squat or lunge exercises using Kbox^®^ (0.10 kg/m ^2^ and 0.05 kg/m^2^), bilateral and unilateral leg-press and leg-curl using Yo-Yo Technology^®^ (0.11 kg/m^2^), several exercises focusing on posterior chain using free-weights and inertial devices (i.e., barbell deadlift, barbell hip-trust, hip-extension or hip-trust in versa pulley^®^), anterior cross chain and posterior cross chain using Kine Dimanics^®^, elastic bands, free-weights and/or body-weight. The main exercises employed in the gym sessions for upper-body, core, and lumbo-pelvic stability were as follows: push-up and pull-up exercises using free-weights and Kine Dynamics^®^, functional bilateral rotational exercises using VP, single leg side, prone and front bridge using Fit ball, cable wood chops using VP and several functional unilateral push and pull exercises using VP+Kine Dynamics^®^.

Specific ST on the field lasted 20–25 min each and consisted of different combined soccer-drills with goal-shooting (finishing), including high-intensity actions such as plyometric jumps, resisted-sprint, duels, different change of directions, or high-speed running, among others.

Activation training consisted of neuromuscular training exercises in the gym or on the field, as an initial part of the training session and before the specific soccer-drills. Examples of exercises employed were as follows: exercises focusing on core, hamstring, groin and abductor muscles activation combined with sensorimotor exercises on stable/unstable surfaces. In addition, some individual activation sessions were also prescribed to some players when the team started directly on the field (∼10 min). Activation-training sessions with the whole group lasted 20 min each.

Individual training consisted of ST sessions in the gym focusing on the player’s weak-points for injury prevention (i.e., imbalances, posterior chain, groin, abductors, calf, and rectus femoris). The individual training was usually planned after the soccer training on the field and lasted 10–15 min.

Players completed several ST sessions during the pre-season: five sessions of ST in the gym, seven sessions of complementary training in the gym, four sessions of specific ST on the field, seven sessions of activations for whole group, and six sessions of individual training. All training sessions were fully supervised by strength and conditioning coaches with the support of physiotherapists, and training diaries were maintained for the whole group. No additional training was allowed. An example of the training programme for the team is shown in Tables 2 and 3.

**Table 2:** An example of typical training schedule during pre-season (one friendly game/week).

	**Monday**	**Tuesday**	**Wednesday**	**Thursday**	**Friday**	**Saturday**	**Sunday**
am	Trip to Training Camp	- **ST GYM^2^** - Individual exercise: individual defensive technique - Group Exercise: specific drill pass - Group Exercise: game possession 4v4 + 3 jokers	- Collective exercise: defensive technique - Group Exercise: specific drill pass & finishing - Collective exercise: game	Rest	- **ST GYM^2^** - Collective exercise: defensive organization - Group Exercise: offensive evolutions - Collective exercise: game	- Group Exercise: generic drill pass - Group Exercise: generic rondos	- Collective & individual recovery
pm	- **Activation^1^** - Collective exercise: individual technique - Group Exercise: generic drill pass - Group Exercise: generic rondos - Group Exercise: game possession 4v4 + 3 jokers	- Group exercise: individual defensive technique - Group Exercise: specific drill pass & finishing - Group Exercise: SSGs 6v6 + Gk - **Complementary ST^3^**	Rest	- ****Introduction HIT: medium distance drill - Group Exercise: offensive evolutions - Collective exercise: conditioned game - **Complementary ST^3^**	- **** Group Exercise: specific drill pass - Collective exercise: offensive organization	***Friendly Game:*** Italian semi-professional team (45 min each player)	Rest

**Notes.**

1 1 × 12 exercises: Russian belt deadlift (8 rep.); Russian belt squat + hip-extension (8 rep.); hip-flexion with band (10 rep./leg); lateral band walk (10 rep./leg); side bridge on fitball (6 rep./side); kneeling on fitball (20 s); single-leg prone bridge on fitball (6 rep/leg); single squat on bosu (8 rep./leg); different landings on bosu (6 rep./leg); lateral lunge on whole body vibration (8 rep./leg); way bunkie side bridge (6 × 6 s./side); skater hops (10 rep.).

2 2 × 12 exercises: single lateral squat on kbox (6 rep./leg); single-leg Yo-Yo Leg-curl (6 rep./leg); anterior-posterior side step on VP (6 rep./leg); lateral crossover cutting on VP (6 rep./leg); trunk rotation on VP+Kine (10 rep./side); single-leg leg press Yo-Yo multigym (6 rep./leg); single standing pull+trunk rotation with kine (10 rep/side); lateral step-up box (8 rep./leg); single-leg dumbbell deadlift hop (8 rep/leg); lateral barbell lunge on whole body vibration (6 rep/leg); downward cable wood chops on VP (8 rep/side); hip-extension on VP (6 rep/leg).

3 2 × 12 exercises: push-up with Kine (12 rep.), pull-up+trunk rotation with Kine (12 rep.), ball pull-over throw (10 rep.), single-arm push+trunk rotation on VP (8 rep./side), single-arm pull on VP (8 rep./side), suspended crunch with Kine (8 rep./side), dumbbell bench press on fitball (12 rep.), cable single-arm row in VP (8 rep./side), seated dumbbell clean on fitball (10 rep.), plank in bench+fitball (20 s.), side plank with fitball (20 s./side), Up-down plank (12 rep.).

### Statistical analysis

Data are presented as mean ± standard deviation (SD). The normality of distribution of the variables were verified using the *Shapiro–Wilk* test. One way analysis of variance (ANOVA) was used to compare variables means. Changes in FFM and FM variables were analysed by two-way repeated measures ANOVA. Post hoc test were calculated with Bonferroni correction for multiple comparison. In addition, possible differences or within-player changes DXA-measured variables were analysed for practical significance using magnitude-based inferences, pre-specifying 0.2 between-subject SDs as the smallest worthwhile effect (Hopkins et al., 2009). The standardized difference or effect size (ES, 90% confidence limit [90%CL]) in the selected variables was calculated. Threshold values for assessing magnitudes of the ES (changes as a fraction or multiple of baseline standard deviation) were <0.20, 0.20, 0.60, 1.2, and 2.0 for trivial, small, moderate, large, and very large respectively (Hopkins et al., 2009). Quantitative chances of higher or lower changes were evaluated qualitatively as follows: <1%, almost certainly not; 1–5%, very unlikely; 5–25%, unlikely; 25–75%, possible; 75–95%, likely; 95–99%, very likely; >99%, almost certain (Hopkins et al., 2009). A substantial effect was set at >75% (Suarez-Arrones et al., 2014; Suarez-Arrones et al., 2015).

**Table 3:** An example of typical training schedule during in pre-season (two games/week).

	**Monday**	**Tuesday**	**Wednesday**	**Thursday**	**Friday**	**Saturday**	**Sunday**
am	- **Activation GYM^1^** - Group Exercise: generic drill pass - Collective exercise: offensive organization + defensive transition - Collective exercise: game possession 9v9 + 3 jokers	- **ST GYM^2^** - Individual exercise: individual technique - Group Exercise: specific drill pass + HIT short intervals drill with COD - Group Exercise: game possession 4v4 + 3 jokers	- Group Exercise: specific drill pass & finishing - Collective exercise: offensive organization + defensive transition - Collective exercise: game **Complementary ST GYM^4^**	Rest	- Players with + 45 min played: - Recovery (aerobic training, Foam Roller, and contrast therapy) Players with - 45 min played: - Group Exercise: SSGs 5v5 + GK + HIT short intervals drill	Rest	Rest
pm	Rest	- **Specific ST Field^3^** - Group Exercise: specific drill pass - Collective exercise: game possession 8v8 + 3 jokers	Rest	*Friendly Game:* Bundesliga Top Team	- Collective exercise: individual technique - Group exercise: generic rondos **Individual training GYM^5^**	** Individual activation** - Group Exercise: specific drill pass - Group Exercise: game possession 4v4 + 3 jokers **Complementary ST GYM**	*Friendly Game:* Bundesliga Top Team

**Notes.**

1 1 × 10 exercises: Russian belt deadlift (8 rep.); Russian belt squat + hip-extension (8 rep.); adductor slides with kine (8 rep./leg); standing abductor with kine (10 s./leg); specific side plank (kicking a ball)(6 rep./side); single-leg plank (10 s./leg); side plank with kine opening and closing legs (6 rep/leg); single balance exercises on bosu (15 s./leg); different landings on bosu (10 rep./leg); frontal lunge on whole body vibration (8 rep./leg).

2 1 × 12 exercises: single lateral squat on kbox + bosu (6 rep./leg); hip-extension kick in Exentrix (6 rep./leg); postero-anterior side step on VP (6 rep./leg); lateral crossover cutting on VP (6 rep./leg); trunk rotation on VP+Kine (10 rep./side); soccer kick exercise with VP (10 rep./leg); single standing with trunk rotation with kine (10 rep/side); step-up box with jump (8 rep./leg); single-leg hamstring bridge on box (5 s. isometric+8 rep/leg); lateral barbell lunge on whole body vibration (6 rep/leg); upward cable wood chops on VP (8 rep/side); landings in bosu with elastic band at the waist (6 rep/leg).

3 Drill 1: 4 × (2 jump hurdle drills + receive and passing the ball + 4 change of directions of 90° + receive the ball + dribbling and finishing. Drill 2: 4 × (20 m sled running with Run Rocket + 30 m of running gradually increasing the speed (>25 km/h) + deceleration + receive the ball + dribbling and finishing . Drill 3: 4×(in pairs, jump up and head the ball + 5 m running pushing between them + 1vs1 and finishing).

4 2 × 12 exercises: push-up+trunk rotation with Kine (12 rep.); pull-up with Kine (12 rep.); push-up + side-plank-leg (12 rep.); single-arm push on VP (8 rep./side); single-arm pull+trunk rotation on VP (8 rep./side); bilateral trunk rotational on VP (8 rep./side); bench press (12 rep.); single-arm dumbbell row (8 rep./side); standing dumbbell fly on fitball (12 rep.), pull up (>6 rep.), bar dip (12 rep.), atomic push-up with kine on bosu (10 rep.).

5 An example of a player: 2 × 6 exercises: hip-extension kick in VP (8 rep/side), single-leg hip thrust with elastic band (8 rep./leg), deadlift (8 rep), walking dumbbell lunge (8 rep.), single-leg Yo-Yo Leg-curl (6 rep./leg), adductor in VP (8 rep./leg).

## Results

### Changes in whole body composition

Changes in BC from the end of the previous season to the beginning of the pre-season (after the off-season period) and toward the end of the RTP (pre-season) are shown in Table 4. FM and %FM substantially increased during the off-season period [*likely* (ES: 0.54 ± 0.49) and *very likely* (ES: 0.87 ± 0.46) changes, respectively], while a substantial decrease was observed after the RTP [*likely* (ES: −0.41 ± 0.24) and *almost certainly* (ES: −0.91 ± 0.40) changes, respectively], reaching values similar to the end of the previous season.

**Table 4:** Changes in BC at the end of the previous season, at the beginning of the pre-season (after the off-season period) and at the end of the retraining period (pre-season). Data are mean ± SD.

**Variables**	**End of the Season (EnS)**	**Beginning of the Pre-season (BP)**	**End of the Training Period (ET)**	**Standardized differences & QA (90% CL) (EnS vs. BP)**	**Standardized differences & QA (90% CL) (EnS vs. ET)**	**Standardized differences & QA (90% CL) (BP vs. ET)**
Body Mass (kg)	73.9 ± 4.4	73.2 ± 5.2	73.8 ± 5.1	−0.14 ± 0.25	−0.04 ± 0.19	0.10 ± 0.15
Fat Mass (kg)	10.8 ± 1.0	11.3 ±1.4^a^	10.7 ±1.2^b^	0.54 ± 0.49 (↑88*/11/1*)	−0.05 ± 0.49	−0.41 ± 0.24 (↓*0/7/92*)^*^
Fat Mass (%)	14.6 ± 0.9	15.5 ±1.4^a^	14.5 ±1.2^b^	0.87 ± 0.46 (↑*99/1/0*)^**^	−0.04 ± 0.51	−0.91 ± 0.40 (↓*0/0/100*)^**^
Fat-free Mass (kg)	63.2 ± 3.9	61.9 ±4.4^a^	63.0 ±4.3^b^	−0.30 ± 0.19 (↓*0/19/81*)^*^	−0.03 ± 0.13	0.27 ± 0.12 (↑*82/18/0*)^*^

**Notes.**

EnS End of the season BP Beginning of the pre-season ET End of the intervention period QA Qualitative assessment CL Confidence Limits

a substantial difference vs. end of the season.

b substantial difference vs. beginning of the preseason.

* *P* < 0.05.

** *P* < 0.01.

FFM was substantially reduced following the DTP [*likely* (ES: −0.30 ± 0.19) changes], while a substantial increase was detected after the RTP [*likely* (ES: 0.27 ± 0.12) changes], reaching values similar to the end of the previous season.

### Changes in arms

Changes in left and right arms from the end of the previous season to the beginning of the pre-season (after the off-season period) and toward the end of the RTP (pre-season) are shown in Table 5. Arms-FM (%) substantially increased during the off-season period [*likely* (ES: 0.42 ± 0.35) and *very likely* (ES: 0.60 ± 0.37) changes, respectively], while arms-FM (%) values returned to values similar to the end of the previous season after the RTP. Statistical analysis showed no changes in arms-FFM.

**Table 5:** Changes in left and right arms at the end of the season, at the beginning of the pre-season (after the off-season period) and at the end of the retraining period. Data are mean ± SD.

***Variables***	**End of the Season (EnS)**	**Beginning of the Pre-season (BP)**	**End of the Intervention Period (ET)**	**Standardized differences & QA (90% CL) (EnS vs. BP)**	**Standardized differences & QA (90% CL) (EnS vs. ET)**	**Standardized differences & QA (90% CL) (BP vs. ET)**
Left Arm Mass (g)	5066 ± 482	5058 ± 778	4848 ±662^a^	−0.01 ± 0.50	−0.41 ± 0.41 (↓*1/17/82*)	−0.40 ± 0.53
Left Arm Fat Mass (g)	733 ± 77	754 ± 118	704 ±90^b^	0.25 ± 0.48	−0.34 ± 0.46	−0.38 ± 0.43 (↓*2/21/78*)
Left Arm Fat Mass (%)	14.5 ± 1.1	15.0 ±1.7^a^	14.6 ± 1.2	0.42 ± 0.35 (↑*86/13/0*)^+^	0.09 ± 0.43	−0.33 ± 0.44
Left Arm Fat-Free Mass (g)	4333 ± 434	4304 ± 700	4288 ± 427	−0.06 ± 0.48	−0.10 ± 0.15	−0.03 ± 0.49
Right Arm Mass (g)	5028 ± 484	5090 ± 777	4936 ± 751	0.12 ± 0.40	−0.17 ± 0.39	−0.29 ± 0.53
Right Arm Fat Mass (g)	740 ± 75	797 ±167^a^	719 ±102^b^	0.70 ± 0.71 (↑*88/9/2*)	−0.25 ± 0.63	−0.43 ± 0.42 (↓*1/16/83*)
Right Arm Fat Mass (%)	14.8 ± 1.3	15.6 ±1.7^a^	14.6 ±0.9^b^	0.60 ± 0.37 (↑*96/4/0*)^*^	−0.10 ± 0.44	−0.53 ± 0.38 (↓*0/7/92*)^*^
Right Arm Fat-Free Mass (g)	4288 ± 444	4293 ± 645	4217 ± 661	0.01 ± 0.34	−0.15 ± 0.33	−0.16 ± 0.43
Left Leg Mass (g)	14211 ± 757	14246 ± 935	14301 ± 817	0.04 ± 0.24	0.11 ± 0.30	0.07 ± 0.37
Left Leg Fat Mass (g)	2141 ± 178	2293 ±246^a^	2230 ±210^a^	0.78 ± 0.50 (↑*97/3/0*)^*^	0.46 ± 0.42 (↑*85/14/1*)	−0.32 ± 0.29
Left Leg Fat Mass (%)	15.1 ± 1.1	16.1 ±1.5^a^	15.6 ±1.3^a^^,^^b^	0.87 ± 0.47 (↑*99/1/0*)^**^	0.44 ± 0.42 (↑*84/15/1*)	−0.32 ± 0.22 (↓*0/17/83*)^*^
Left Leg Fat-Free Mass (g)	12070 ± 678	11953 ± 835	12208 ±819^b^	−0.16 ± 0.20	0.19 ± 0.21	0.28 ±0.24^+^
Right Leg Mass (g)	14396 ± 628	14484 ± 702	14748 ± 856	0.13 ± 0.38	0.51 ± 0.37	0.38 ± 0.47
Right Leg Fat Mass (g)	2154 ± 145	2358 ±264^a^	2272 ±251^a^	1.29 ± 0.78 (↑*98/1/0*)^*^	0.74 ± 0.67 (↑*91/7/1*)	−0.30 ± 0.44
Right Leg Fat Mass (%)	15.0 ± 1.1	16.3 ±1.6^a^	15.4 ±1.4^b^	1.13 ± 0.49 (↑*100/0/0*)^**^	0.36 ± 0.51	−0.51 ± 0.37 (↓*0/8/92*)^*^
Right Leg Fat-Free Mass (g)	12241 ± 604	12126 ± 637	12476 ±741^a^^,^^b^	−0.17 ± 0.23	0.35 ± 0.32 (↑*80/19/1*)	0.50 ± 0.34 (↑*93/7/0*)^*^
Trunk Mass (g)	35223 ± 2698	34363 ± 2791	34917 ± 2575	−0.29 ± 0.29	−0.10 ± 0.25	0.19 ± 0.21
Trunk Fat Mass (g)	4999 ± 637	5135 ± 731	4793 ±688^b^	0.20 ± 0.39	−0.30 ± 0.46	−0.49 ± 0.25 (↓*0/3/97*)^*^
Trunk Fat Mass (%)	14.2 ± 1.2	14.9 ±1.5^a^	13.7 ±1.3^b^	0.55 ± 0.39 (↑*93/6/0*)^*^	−0.36 ± 0.47	−0.91 ± 0.31 (↓*0/0/100*)^**^
Trunk Fat-Free Mass (g)	30224 ± 2298	29228 ±2346^a^	30123 ±2067^b^	−0.40 ± 0.26 (↓*0/10/90*)^*^	−0.04 ± 0.19	0.36 ± 0.21 (↑*89/11/0*)^*^

**Notes.**

EnS End of the season BP Beginning of the pre-season ET End of the intervention period QA Qualitative assessment CL Confidence Limits

a substantial difference vs. end of the season.

b substantial difference vs. beginning of the preseason.

+ *P* = 0.06.

* *P* < 0.05.

** *P* < 0.01.

### Changes in Legs

Changes in left and right legs from the end of the previous season to the beginning of the pre-season (after the off-season period) and toward the end of the RTP (pre-season) are shown in Table 5. Legs-FM (%) substantially increased during the off-season period [*very likely* (ES: 0.87 ± 0.47) and *almost certainly* (ES: 1.13 ± 0.49) changes, respectively], while after the RTP legs-FM (%) values returned to values similar to the end of the previous season. Statistical analysis showed no changes in legs-FFM after the off-season period, with a substantial increase in legs-FFM after the RTP in comparison with the beginning of pre-season [*likely* (ES: 0.50 ±0.34) and *trend* (ES: 0.28 ± 0.24) changes], and a trend to increase in right-leg FFM in comparison with the end of the previous season [*likely* (ES: 0.35 ± 0.32) changes].

### Changes in trunk

Changes in trunk from the end of the previous season to the beginning of pre-season (after the off-season period) and toward the end of the RTP are shown in Table 5. Trunk-FM (%) substantially increased during the off-season period [*likely* (ES: 0.55 ± 0.39) changes], while after the RTP the FM (%) values returned to values similar to the end of the previous season. Trunk-FFM substantially decreased during the off-season period [*likely* (ES: −0.40 ± 0.26) changes], while the FFM values after the RTP returned to values similar to the end of the previous season.

## Discussion

The present study analysed the effects of a five-week DTP with an individual training program prescribed to each player, and six and a half weeks of RTP combining soccer and ST on FFM and FM in elite professional male soccer players. The main findings of the present study were that: (i) whole body %FM increased and FFM reduced after DTP, returning to values comparable to the end of the previous season after RTP; (ii) arms, legs, and trunk %FM increased after DTP, returning to the values observed at the end of the previous season after RTP; (iii) trunk-FFM was reduced after DTP, returning to the initial values after RTP; and (iv) legs-FFM did not change after DTP, with a substantial increase after RTP in comparison with pre-season values.

The BC of soccer players is likely to change during the course of the competitive season as a result of training, detraining, competition, and/or diet (Iga et al., 2014). In the group of football players investigated in the present study, DXA-derived measurements showed a whole-body increase in %FM (5.9%, small ES) during the off-season. The %FM at regional level also substantially increased after DTP, distributed in the trunk, upper body, and lower body. At the whole-body level, DXA-measured data indicated slightly higher %FM increments (7.3%) in elite professional soccer players after a DTP in which athletes avoided physical activity for at least three weeks, with only one week of planned aerobic running training (Reinke et al., 2009). In supporting to our findings, increases in FM assessed by skinfold thickness measurements, were also found by Koundourakis et al. (2014) and Sotiropoulos et al. (2009) in professional soccer players after a transition period (6 vs. 4 weeks, respectively) with a training program that in both cases included four weeks of low intensity aerobic running (Koundourakis et al., 2014), and aerobic and strength conditioning activities (Sotiropoulos et al., 2009), respectively. Similar results using skinfold thickness were also shown with elite professional players from the first division Spanish League (9.5%) after a seven-week DTP including four weeks of aerobic training and ST (Requena et al., 2017). In contrast, a recent study (Joo, 2018) showed no changes in %FM, assessed by bioelectrical impedance analysis (InBody 520), after a detraining and retraining period during the off-season. Previous studies (Munguia-Izquierdo et al., 2018; Suarez-Arrones et al., 2018a) demonstrated that bioelectrical impedance analysis of %FM data showed an unclear relationship with DXA (*r* = 0.36), with *almost certainly* differences (large ES). Based on these studies (Munguia-Izquierdo et al., 2018; Suarez-Arrones et al., 2018a), bioelectrical impedance analysis could be considered inappropriate for estimating %FM in professional soccer players, which may explain the difference in the results obtained.

The results of the present study showed a substantial decrease in %FM after the RTP during the pre-season (−5.9%), returning to values similar to the end of the previous season. In the same line and also using DXA, Reinke et al. (Reinke et al., 2009) revealed a decrement in %FM during the pre-season training in soccer players from the German Bundesliga in comparison with the end of the off-season period (−14.5%). Contrary to our results, Carling & Orhant (Carling & Orhant, 2010) in a study with elite professional soccer players of the French League 1, showed no changes in %FM after the pre-season training. Of note, these authors used skinfold measurements to estimate %FM. Neither of the two previous studies (Carling & Orhant, 2010; Reinke et al., 2009) compared the pre-season and in-season values with those found at the end of the preceding season. In soccer, an excess of adipose tissue loads the player with useless extra weight (Kraemer et al., 2004) and likely contributes to greater energy expenditure during a match and impaired power and acceleration performance (Rico-Sanz, 1998). Therefore, to enhance soccer performance, players need to begin the in-season period (competition) in the best possible condition for success, with optimal levels of FM.

In the present study, whole-body data by DXA showed substantial decreases in FFM (−2.1%) after the off-season period. Similar results were found by Reinke et al. (Reinke et al., 2009) after a DTP avoiding physical activity for at least three weeks, with a significant reduction in lean mass (−3%). When FFM was evaluated by body segments, our results only detected decreases in the arms and trunk regions, while lower limb FFM after the DTP remained unchanged. No previous studies have included information about FFM evaluated by body segments using DXA after a DTP with professional soccer players. Requena et al. (Requena et al., 2017), employing skinfold thickness, showed a reduction in FFM in lower-limbs and no changes in upper-body after seven weeks of DTP including four weeks of aerobic and ST in elite professional soccer players. Decreases in lean-mass and muscle strength deficiency are believed to be one of the main risk factors associated with muscle strain injuries in soccer (Croisier et al., 2008; De Hoyo et al., 2014; Mendez-Villanueva et al., 2016; Timmins et al., 2016), and there is a relationship between high force production capabilities of soccer players and reduced post-match markers of muscle damage (Owen et al., 2015). In order to avoid these decreases in lean mass and potentially muscle strength, and/or physical fitness, strength and conditioning coaches design specific training programs for the off-season period (Requena et al., 2017), as has been the case in the present study. Based on our results, our data revealed that players maintained lower-limb FFM after the DTP. We can hypothesize that this positive result was probably due to the lower-limb ST program and the players’ compliance rate, in conjunction with the extensive previous experience of the players in lower-limb eccentric-overload training using flywheel devices throughout the previous season.

Whole-body data demonstrated substantial increases in FFM (2%) after the RTP during the pre-season phase. Reinke et al. (Reinke et al., 2009) presented similar results using DXA at the end of five-weeks pre-season training (2.3%), with two to three sessions per day, alternating high-intensity endurance and power training with football skills. Contrary to our results, Carling & Orhant (Carling & Orhant, 2010) reported no changes in FFM at the end of a pre-season period (using skinfold measurements). When FFM was evaluated by body segments, our results showed increases in lean mass in the trunk (3%) and lower-limbs (2.5%) at the end of the RTP, during pre-season. No previous studies have included information about the changes in FFM by body segments using DXA after a pre-season in professional soccer players. The substantial increase in trunk FFM found in the present work after the RTP is an interesting finding, since preventive neuromuscular training programs with strength and proximal control training demonstrated the greatest prophylactic effects on ACL injury risk reduction (Sugimoto et al., 2015), and recent studies identified a link between proximal segment control and knee joint injury (Hewett, Torg & Boden, 2009; Zazulak et al., 2007). In addition, players who sustained severe ligamentous knee injuries demonstrated greater deficits in trunk neuromuscular control (Zazulak et al., 2007). Thus, upper-body and core strength and control can be regarded as important physical qualities in professional football, being an integral part of an effective injury prevention program (Melegati et al., 2013; Owen et al., 2013) and facilitating abilities such as sprinting, jump, agility, or change of direction, having a high impact on all types of duels and contact actions during match-play. In the same line, the substantial increase in lower-limb FFM found in the present work after the RTP is an important result. Neural and hypertrophic adaptations are the basis of muscle strength development and their respective mechanism in the neuromuscular system are distinct (Silva, Nassis & Rebelo, 2015; Toigo & Boutellier, 2006). Although we did not evaluate muscle strength, previous studies have shown that the neural factor accounts for early strength gains while the hypertrophic factor has a later onset (Sale, 1988; Seynnes, Boer & Narici, 2007). Improving the strength of soccer players does not necessarily imply an increase in lean mass (Toigo & Boutellier, 2006) and the relative increase in muscle strength is greater than could be accounted for by the increase in muscle volume (Tesch et al., 2004). Therefore, the greater lower limb lean mass and, possibly, muscular strength in our players at the beginning of the competition period will help players to reduce muscular damage following match-play and the muscle strain injury rate during competitions (Owen et al., 2015; Timmins et al., 2016).

This work has some limitations that should be acknowledged. First, the small sample size was composed of professional players from an elite professional club during a DTP and RTP. Therefore, present findings might not apply to other soccer populations (e.g., youth, female). Second, the determinant influence of diet on BC was not controlled exhaustively throughout the DTP. It would be interesting for future investigations to completely control these variables. Our study assessed players from an elite professional football club, and this group of participants has not allowed us to have a control group (professional ethics).

## Conclusion

In conclusion, 3 weeks of the planned individual training program during the five-week DTP resulted in a small increase in FM in whole body and regional segments along with a small decrease in trunk FFM, with no changes in lower-limb FFM. Due to the intensified ST program carried out in conjunction with the football training during the pre-season, %FM and trunk FFM values returned to levels previously observed in the preceding season, and a small increase in lower-limb FFM was found in comparison with the beginning of pre-season. These results suggest that elite soccer players can be allowed 2 weeks of rest during a five-week DTP, since the changes in %FM and FFM were relatively small and FM and FFM returned to the optimal initial values for competition after the proposed RTP.

##  Supplemental Information

10.7717/peerj.7466/supp-1 Supplemental Information 1 Raw Data from DXA

## Additional Information and Declarations

### Data Availability

The following information was supplied regarding data availability: The raw data measurements are available in a Supplemental File.
